# Vaccine stockpile sharing for selfish objectives

**DOI:** 10.1371/journal.pgph.0001312

**Published:** 2022-12-05

**Authors:** Shashwat Shivam, Joshua S. Weitz, Yorai Wardi

**Affiliations:** 1 School of Electrical and Computer Engineering, Georgia Institute of Technology, Atlanta, GA, United States of America; 2 School of Physics, Georgia Institute of Technology, Atlanta, GA, United States of America; 3 School of Biological Sciences, Georgia Institute of Technology, Atlanta, GA, United States of America; 4 Institut de Biologie, École Normale Supérieure, Paris, France; CSIR-Indian Institute of Chemcial Technology, INDIA

## Abstract

The COVAX program aims to provide global equitable access to life-saving vaccines. Despite calls for increased sharing, vaccine protectionism has limited progress towards vaccine sharing goals. For example, as of April 2022 only ~20% of the population in Africa had received at least one COVID-19 vaccine dose. Here we use a two-nation coupled epidemic model to evaluate optimal vaccine-sharing policies given a selfish objective: in which countries with vaccine stockpiles aim to minimize fatalities in their own population. Computational analysis of a suite of simulated epidemics reveal that it is often optimal for a donor country to share a significant fraction of its vaccine stockpile with a recipient country that has no vaccine stockpile. Sharing a vaccine stockpile reduces the intensity of outbreaks in the recipient, in turn reducing travel-associated incidence in the donor. This effect is intensified as vaccination rates in a donor country decrease and epidemic coupling between countries increases. Critically, vaccine sharing by a donor significantly reduces transmission and fatalities in the recipient. Moreover, the same computational framework reveals the potential use of hybrid sharing policies that have a negligible effect on fatalities in the donor compared to the optimal policy while significantly reducing fatalities in the recipient. Altogether, these findings provide a self-interested rationale for countries to consider sharing part of their vaccine stockpiles.

## Introduction

COVID-19 vaccines are effective in reducing severe infections, hospitalizations and fatalities [1, 2], however, the availability of doses across countries remains highly non-uniform [3]. While sharing vaccines between countries has been termed a global imperative [4–7], many countries have not initiated vaccine sharing campaigns or have initiated limited campaigns that could promote vaccine equity worldwide. Vaccine sharing is expected to reduce the intensity of outbreaks in recipients while reducing the risk of case importation when stringent travel regulations are relaxed in a donor country [8–10]. However, vaccine sharing comes with a potential cost: reducing the availability of vaccines for the donor. This potential cost may also undermine public support for vaccine sharing campaigns. For example, one US-based survey revealed that the majority of individuals prefer that the US donates 10% or less of COVID-19 vaccine stockpile to recipient countries [11]. This perception may increase resistance to vaccine sharing campaigns at national scales.

Epidemic models can be used as part of efforts to assess the costs and benefits of vaccine stockpile sharing between countries. A recent paper [12] studied the efficacy of vaccine sharing between two countries with extensive travel between the donor and recipient. Epidemic dynamics within each country were coupled through the occasional importation of cases and/or the travel of individuals to and from the donor to the recipient (where they could be infected) and back again (where they could accelerate disease transmission). This study concluded that the greatest reduction in fatalities within a focal donor occurred in the absence of sharing. This epidemic model analysis included a time-dependent sharing protocol while assuming that vaccination rates were high, e.g., the eligible population would be fully vaccinated within 50 days. Related efforts evaluated the question of optimal vaccine sharing after a donor is already at or near herd immunity [13]. In that case, donating ‘surplus’ vaccines to the group of COVAX countries can reduce fatalities within the recipient and donor countries. However, as the donor is near herd immunity, the epidemic is already in decline such that sharing leads to negligible reductions in infections in the donor country. Both modeling studies [12, 13] make the implicit assumption that vaccination rates in donor countries are fast enough so that the population in the donor rapidly reaches herd immunity. However, in practice, vaccination coverage has increased far slower than anticipated, e.g., in the US, one year after eligibility for vaccinations were extended to all adults above 16, only 67% of adults had been vaccinated [14]. This failure to rapidly vaccinate populations in vaccine-rich countries raises new questions on how to optimally utilize vaccine stockpiles to reach the objectives—specifically is it in a country’s ‘self-interest’ to donate and, how do optimal policies vary when vaccination rates are low?

In this manuscript, we consider a two-country epidemic model in which a donor country can share vaccines with a recipient country. In doing so, we seek the optimal sharing policy given a selfish objective. The term ‘selfish objective’ refers to a policy of reducing total national fatalities in the donor, regardless of the effect on the recipient. Further, we assume that the population is immunologically naive—given our intent to retrospectively assess early decision-making and to potentially inform decision making in the future. In the present analysis, we consider a broader range of vaccination rates than has been considered in related studies. This choice is motivated by realized coverage rates from the USA (where ~67% of the population was fully vaccinated in a period of 1 year) and UK (~69% were fully vaccinated in 1 year) [15]. As we show, epidemic model analysis reveals a broad range of vaccine uptake rates and cross-nation epidemic coupling rates in which the optimal self-interested policy is for the donor to share a substantial fraction of its vaccine stockpile with a recipient. Based on our model, we show sharing vaccines can reduce the fatalities in the donor country with the added benefit of curbing the spread of COVID-19 in the recipient country.

## Vaccine sharing problem

We consider two countries, A and B, each confronting a COVID-19 outbreak in which one country (A—the donor country) has a vaccine stockpile and the other country (B—the recipient country) does not. The outbreak is modelled using SEIRV (Susceptible—Exposed—Infected—Recovered—Vaccinated) dynamics. The epidemic dynamics of the countries are coupled such that active infections in one can cause infections in the other. Further, A has the option of donating a part of its vaccine stock to B. In our search for optimal stockpile sharing strategies, we assume that sharing is done once, before a vaccination campaign begins in either the donor or recipient country.

The objective of the vaccine sharing policy is to minimize fatalities in A, in the event that the epidemic dynamics of donor and recipient countries are coupled. Here, we explore optimal policies as a function of epidemiological parameters as well as two features of the two-nation problem: (i) the epidemic-coupling constant; (ii) the rate of vaccine uptake in the donor country. The epidemic-coupling constant controls the likelihood that infections in the recipient country lead to new cases in the donor country (and vice-versa). The vaccine uptake rate controls the rate at which a donor country can potentially use its vaccine stockpile. Given slow vaccine coverage rates [14], we focus on limits in which the rate of vaccine uptake (on the order of a year) is slower than that of typical strain-level outbreak dynamics (i.e., on the order of months). In all cases the objective of the donor is to minimize fatalities in its own country. Later we relax this assumption to propose near-optimal solutions that result in substantially-lower mortality rates in the recipient with only small increases in fatalities experienced by the donor. The details of the dynamic model and related optimization problem are available in the Methods section.

## Results

**Fig 1:**
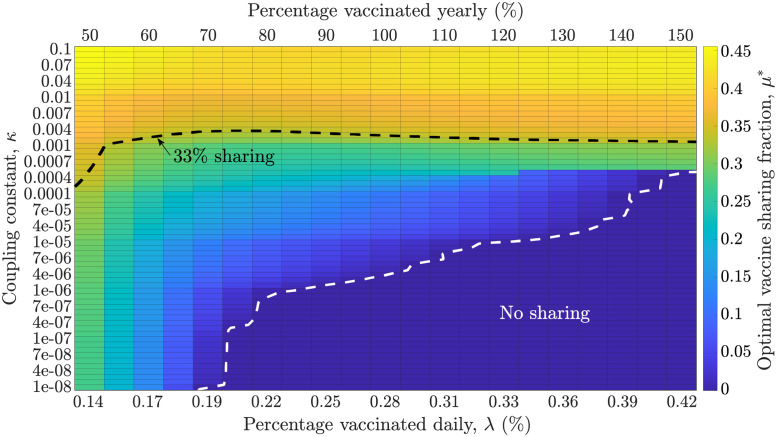
Dependence of the optimal fraction of vaccines (μ*) donated as a function of the vaccination rate, λ (x-axis) and the epidemic coupling coefficient, *κ* (y-axis). For very low values of *κ* and moderate to high vaccination rates, the optimal sharing fraction is 0. For all other values, the optimal vaccination donation fraction from donor to recipient is positive, increasing with the epidemic coupling constant and the realized vaccination rate in country A. The black dashed line shows a level curve of μ* = 1/3, while the white dashed line demarcates the region where μ* = 0, that is, there is no vaccine sharing.

We consider the two countries to have an initial population of 10^7^ with 500 initial infections in which country A has enough vaccines to fully vaccinate 7 × 10^6^ individuals. The optimal vaccine sharing fraction μ* is the value which minimizes the total fatalities in A over a given time horizon, regardless of its effect on B. We explore the dependence of the optimal policy with respect to the daily vaccination rate, λ, and the epidemic coupling constant, *κ*. Simulation results are contained in the heatmap shown in Fig 1. If the vaccination rate is sufficiently high, then for low epidemic coupling *κ* ≤ 10^−6^, the optimal vaccine sharing fraction is 0. These findings are consistent with the results reported in [12]—the rationale is that from the perspective of the optimization criterion it is more effective to rapidly vaccinate nearly the entire population in county A than to share and reduce the importation of cases from a largely disconnected recipient country. However, when vaccination rates within A are low (e.g., less than 0.19%/day, equivalent to 70%/year), then the optimal vaccine sharing fraction is positive for all epidemic coupling constants studied. When the epidemic coupling constant between the two countries is stronger, then it is beneficial to donate more vaccines to country B. The optimal donation fraction is negatively correlated with the vaccination rate (see Fig 1). Moreover, we also find a critical transition: if the vaccination rate is low, then the optimal policy is to share vaccines with country B even for very low epidemic coupling constant values. We interpret this finding to mean that sharing vaccines reduces epidemic intensity in a recipient nation, thereby reducing case importation to the donor nation. In effect, sharing provides greater benefits, particularly when vaccine uptake rates in donor nations are sufficiently slow that the vaccines would be otherwise ‘wasted’.

**Fig 2:**
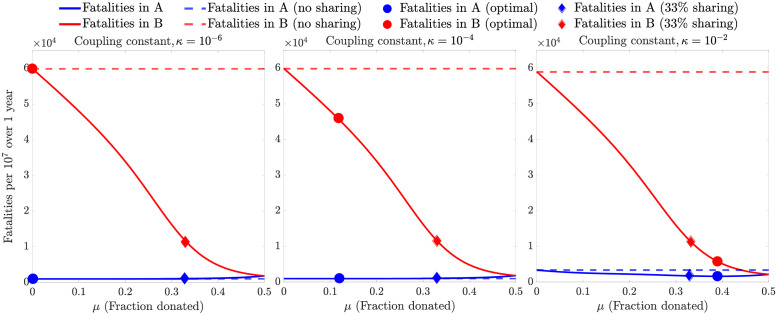
Model-based fatalities per 10^7^ over 1 year in countries A and B for different values of vaccines donated from A to B (*μ*). The value of *μ* which minimizes the fatalities in country A is termed the ‘optimal policy’. The simulation is run for low, medium and high epidemic coupling constants, *κ* (with *κ* ∈ {10^−6^, 10^−4^, 10^−2^}) and the daily vaccination rate is held fixed at 0.277% of the total population in all three panels. The optimal fraction for every *κ* is marked on the corresponding plot with a circle, along with the fatalities in countries A and B for a 33% sharing policy (diamond) and the no-share policy (dashed line).

Fig 1 provides the optimal vaccine sharing fraction for different parameter combinations. Next, we explore the impact of implementing optimal sharing policies on epidemic outcomes. To do so, we quantify the fatalities in A and B when no vaccines are shared (*μ* = 0) and compare these baseline levels of fatalities to simulated epidemic outcomes for different values of *μ*—including the optimal levels μ*. Fig 2 shows the deaths per 10^7^ individuals in both countries for different values of *μ*, for three values of the epidemic coupling constant *κ* (low, medium and high), with a fixed daily vaccination rate of 0.277% of the total population. This vaccination rate is equivalent to vaccinating 50% of a country in 6 months. For the three values of *κ*, sharing a small fraction of the vaccines (*μ* > 0) rapidly reduces the fatalities in B and either decreases the fatalities in A (*κ* = 10^−2^) or has a negligible adverse effect (*κ* = 10^−4^, 10^−6^). For *κ* = 10^−6^, the optimal sharing fraction is μ* = 0, hence the optimal policy is the same as the no-share policy. For *κ* = 10^−4^, the fatality reduction in A is negligible, but there is a ~24 reduction in fatalities in B given an optimal sharing fraction of ~0.11>. Lastly, for *κ* = 10^−2^, the optimal policy is to share ~0.38, or more than one-third of the donor’s vaccine stockpile. Compared to the no-share policy (*μ* = 0), there is a ~50% and ~90% reduction in fatalities in countries A and B respectively. By donating a part of the vaccine stock to B, the infections of individuals in country B are reduced which in turn reduces the cross-infections of individuals in country A. However, this level of vaccine sharing also reduces the fraction of the population of country A which is vaccinated. The magnitude of these competing factors is dependent on the epidemic coupling constant *κ*, which explains the shift towards a higher sharing policy as *κ* increases.

**Fig 3:**
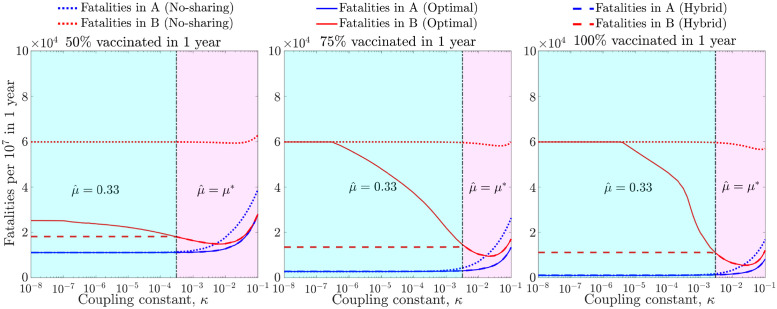
Fatalities per 10^7^ over 1 year for the no-sharing, optimal and hybrid policies in countries A and B given different epidemic coupling constants (*κ* ∈ [10^−8^, 10^−1^]) and three vaccination rates (50%, 75% and 100% of the population vaccinated in 1 year). The hybrid policy maintains a vaccine sharing fraction of 0.33 to the left of the vertical black line, where the optimal vaccine sharing fraction is lower than 0.33 (shown here in cyan). To the right, the optimal vaccine sharing fraction is equal or greater than 0.33, so the hybrid vaccine sharing fraction is equal to the optimal in this region (shown here in magenta). The interpretation of the curves is same for each of the panels: blue denotes fatalities in A and red denotes fatalities in B. For each, the results are shown for no-sharing (dotted), optimal sharing (solid) and hybrid (dashed).

This optimal-sharing analysis reveals that small deviations from the optimal policy leads to small changes in fatalities in A in both the high and medium epidemic coupling scenarios (see Fig 2). Hence, we explore a hybrid policy, which is the same as the optimal policy when μ* is high, and has a fixed, non-zero vaccine sharing fraction when μ* is low—we consider a 1/3 sharing fraction as an illustrative policy heuristic. Denoting by $\hat{\mu }$ the vaccine-sharing fraction resulting from this policy, we define it as $\hat{\mu }=\text{max}\{\mu^{*},1/3\}$. To examine the efficacy of the optimal and hybrid policies, we compare the reduction in fatalities in countries A and B using these policies against the no-sharing policy. Fig 3 compares the hybrid, optimal and no-sharing policies, revealing that the hybrid policy increases fatalities in A by less than 10% when the vaccination rate is high (≥75%) and the epidemic coupling constant is low. However, for these scenarios, there is a large reduction in fatalities in B (≥70%), which is greater than the corresponding reduction achieved by the optimal policy. Thus, by slightly relaxing the requirement of having a policy designed only for minimizing fatalities in A, vaccine sharing can lead to dramatic changes in fatalities in the recipient country with limited impacts on the donor country. Similar results are found in a continuum of scenarios when varying both the vaccine uptake rate and the epidemic coupling constant (see S1 and S2 Figs).

## Discussion

In order to evaluate the impact of vaccine sharing on epidemic outcomes, we explored the optimal sharing policy between a donor country and a recipient country given coupled epidemic dynamics. Given the objective of minimizing fatalities in the donor country, simulation of epidemic dynamics reveal that the optimal policy is for the donor country to share vaccines across a broad range of realistic vaccination rates. This effect is intensified as epidemic coupling between countries increases. These results suggest that the objective of minimizing national fatalities in a donor can nonetheless lead to increased vaccine sharing. Furthermore, by donating vaccines the outbreak and deaths in a recipient can be reduced significantly.

The optimal policy of vaccine allocation is dependent on the epidemic coupling constant and the daily vaccination rates. We find that the optimal sharing fraction is 0 in the regime of high vaccination rates and low-to-moderate epidemic coupling, consistent with related findings [12]. The reason is that if a nation has a large stockpile and can both isolate itself and vaccinate its population at a fast rate, its optimal strategy could be to use its vaccines for developing immunity in its population. However, under conditions of lower vaccination rates, the optimal policy may shift to vaccine sharing. We interpret this finding as follows: when vaccination rates are low and stockpiles are high, then a donor country is not effectively using its vaccine. Instead, a large part of its population could become infected before they can get vaccinated. Therefore donor countries have a dynamical incentive to share vaccines with a recipient country. Vaccination in a recipient country reduces transmission and, in turn, reduces cross-national infection. We note that vaccine sharing remains optimal even in the limit of very low coupling when vaccination rates are sufficiently low, i.e., the dynamic benefits of sharing outweigh the benefits of holding on to a vaccine stockpile that is used too slowly, or not at all.

The finding that minimizing fatalities in donor country can lead to vaccine sharing policies can be extended to alternative, near-optimal policies. In this paper, we proposed and analyzed a hybrid policy that involves sharing vaccines (e.g., 1/3 of the vaccine stock) even when the optimal self-interested policy is not to share. As vaccination rates decrease, this policy can result in negligible impacts on fatalities in the donor country while leading to significant reductions in fatalities in the recipient country. Future work could aim to incorporate the benefits and feasibility of a hybrid policy by a donor country, and its impact on both transmission rates and the evolution of new strains.

The model used for simulating the epidemic and evaluating vaccine sharing policies comes with caveats. The effects of infections in one country on another is modeled and simulated through the use of an epidemic coupling constant rather than through more complex travel policies (e.g., [16–18]). In searching for optimal sharing policies, we assume that both countries have equal vaccination rates given available stockpiles and similar efficiency of isolating infected cases. In practice, preexisting disparities in vaccine access are likely to extend to disparities in the efficacy of vaccination campaigns, case tracing and isolation. As a result the benefits of vaccine sharing to a recipient nation are more likely to be accrued if they are accompanied with accompanying investments in public health infrastructure [19]. Finally, we have assumed that immunity does not wane over the time-scale of the optimization (i.e., approximately 1 year). Future work should consider the impact of waning immunity, due to a combination of changes in immunological status in individuals and the impact of new variants, including immune escape variants [20–22].

In summary, the optimization framework developed in this paper demonstrates the potential value of vaccine-sharing for minimizing national fatalities in donor countries. We find broad regimes of vaccination rates and epidemic coupling constants, where sharing vaccines is both optimal for the donor country and leads to significant reductions in fatalities in both the donor and recipient countries. Moving forward, it will be essential to evaluate extensions of the present framework to more realistic models, including the challenge of sharing campaigns involving multiple donor and recipient countries.

## Methods

The dynamic model consists of a SEIRV framework for each one of the two considered countries, connected by a force of infection describing the crossover rate of infection. The population of country *i* (*i* ∈ {*A*, *B*}) is divided into the following groups: susceptible, exposed, infectious, recovered, and vaccinated. The respective sizes of these groups are denoted by *S*_*i*_, *E*_*i*_, *I*_*i*_, *R*_*i*_, and *V*_*i*_, and additionally, the total number of fatalities is denoted by *D*_*i*_. The population vector of country *i* is denoted by *P*_*i*_ ≔ [*S*_*i*_, *E*_*i*_, *I*_*i*_, *R*_*i*_, *V*_*i*_]^⊤^. Let *c*_*S*_, *c*_*E*_, *c*_*I*_, *c*_*R*_ and *c*_*V*_ denote the respective contact rates of the subpopulations *S*_*i*_, *E*_*i*_, *I*_*i*_, *R*_*i*_ and *V*_*i*_ in country *i*, where it is assumed for the sake of simplicity of presentation that each one of these quantities is identical for both countries. Define *c* as the vector of contact rates, *c* = [*c*_*S*_, *c*_*E*_, *c*_*I*_, *c*_*R*_, *c*_*V*_]^⊤^. Furthermore, let *η*_*I*_ be the measure of disease transmission effectiveness from an infectious person to an exposed individual [23], and let *κ* be the epidemic coupling constant capturing the reduced contact rate between the populations in countries A and B.

The dynamics of the two countries are coupled through their *force of infection* defined, in country *i*, as

$$\begin{array}{c} F\left(P_{i},P_{j},\kappa_{i}\right)=\eta_{I}c_{S}[\frac{c_{I}\left(I_{i}+\kappa I_{j}\right)}{c^{T}\left(P_{i}+\kappa P_{j}\right)}+\kappa \frac{c_{I}\left(I_{j}+\kappa I_{i}\right)}{c^{T}\left(P_{j}+\kappa P_{i}\right)}]; \end{array}$$ (1)

here the index *j* signifies the complement of *i* in the set {*A*, *B*}. An alternate approach in [18] considered the risk of case importation using travel data. The term $\frac{c_{S}c_{I}(I_{i}+\kappa I_{j})}{c^{T}(P_{i}+\kappa P_{j})}$ in the Right-Hand Side (RHS) of Eq. (1) is the probability that a susceptible individual from country *i* comes in contact with an infected individual in country *i* regardless of the latter’s nationality; note the role of *κ* in attenuating *I*_*j*_. Similarly, the term $\kappa \frac{c_{S}c_{I}(I_{j}+\kappa I_{i})}{c^{T}(P_{j}+\kappa P_{i})}$ in the RHS of Eq (1) represents the probability that a susceptible individual from country *i* comes in contact with an infected individual at country *j* regardless of the origin of the latter.

This force of infection extends the standard notion associated with a single country [24]. To see this, suppose that all the contact rates are equal to a baseline contact rate, *c*_*B*_, and *κ* = 0, namely the pandemics in the two countries are decoupled. Assuming relatively few fatalities per capita, the total population is approximately constant *N*, hence the force of infection can be approximated by *F* ≈ *η*_*I*_*c*_*B*_(*I*/*N*); this gives the standard SEIR model by defining *β* = *ηc*_*B*_ as the *baseline infection rate*.

To determine a reasonable range of values for *κ*, we examine the total number of inbound travellers to a country in 2020. Specific estimates of *κ* for country pairs connected predominantly via air travel can be computed via passenger tracking data. as in [17]. Here, we use aggregate air travel data as a means to develop an initial estimate of *κ*, while noting that the actual number of travellers between any two countries is highly heterogeneous. With this assumption, considering a world population of 7.770 billion in 2020 [25], and 19 million, 12 million, and 7 million air travelers in USA, Germany, and Greece respectively [26–28], we can estimate the fraction of the total number of individuals arriving from a country with a population of 10 million. Next, we estimate the probability of coming in contact with a traveller versus the probability of contacting a resident, based on the ratio of person-days of visitors to that of residents. For example, in the case of the USA with a population of 330 million [29], ~19 million travellers in 2020 and an average stay duration of 18 days [30], we get a value of $\kappa =\frac{(19\times 10)/7770}{330}\times \frac{18}{365}\approx 3.7\times 10^{-6}$. For Germany, with a population of 84 million [31], ~12 million visitors in 2020 and an average stay duration of 12 days [32], we get $\kappa =\frac{(12\times 10)/7770}{84}\times \frac{12}{365}\approx 6\times 10^{-6}$. For Greece, with a population of 11 million [33], ~7 million visitors in 2020 and an average stay duration of 9 days [34], we get $\kappa =\frac{(7\times 10)/7770}{11}\times \frac{9}{365}\approx 2\times 10^{-5}$. In practice, we expect significant variability when considering a specific country pair. Thus, we study the epidemic behavior for *κ* values ranging both above and below these central tendencies, i.e., we explore ranges of *κ* from 10^−8^ to 10^−1^.

Returning to the case discussed in this paper, assume that a person can be vaccinated only if in the susceptible state, and denote by λ_*i*_ the daily vaccination rate in country *i*. We assume that λ_*i*_ is dependent on vaccine availability in the following way. Given a constant λ > 0 representing the operational vaccination rate in either country, λ_*i*_ is defined as

$$\begin{array}{c} \lambda_{i}=\{\begin{array}{cc} \lambda , & \;\;\;\text{if}\;\text{the}\;\text{vaccine}\;\text{stockpile}\;\text{is}\;\text{not}\;\text{empty}\\ 0, & \;\;\;\text{if}\;\text{the}\;\text{vaccine}\;\text{stockpile}\;\text{is}\;\text{empty}; \end{array} \end{array}$$

note that λ is assumed to be independent of the specific country where the vaccination is performed. Let the total vaccine-stock available before sharing be *V*_0_. At the start of the simulation, country A donates a fraction of its vaccine stock to B, denoted by *μ* ∈ [0, 1]. Let $V_{i}^{ini}$ denote the initial vaccine stock in country *i*, thus $V_{A}^{ini}=(1-\mu )V_{0}$ and $V_{B}^{ini}=\mu V_{0}$. Furthermore, let *T*_*inf*_ be the infectious period; *T*_*inc*_ be the disease incubation period; and *φ* be the case fatality ratio for infected individuals. In the present case, the model for country *i* is given by the following system of differential equations,

$$\begin{array}{c} \begin{array}{cc} {\dot{S}}_{i} & =-F\left(P_{i},P_{j},\kappa \right)S_{i}-\lambda \left(V_{i}^{ini},S_{i}\right)\\ {\dot{E}}_{i} & =F\left(P_{i},P_{j},\kappa \right)S_{i}-\frac{1}{T_{inc}}E_{i}\\ {\dot{I}}_{i} & =\frac{1}{T_{inc}}E_{i}-\frac{1}{T_{inf}}I_{i}\\ {\dot{R}}_{i} & =\left(1-\varphi \right)\frac{1}{T_{inf}}I_{i}\\ {\dot{V}}_{i} & =\lambda (V_{i}^{ini},S_{i})\\ {\dot{D}}_{i} & =\frac{\varphi }{T_{inf}}I_{i}. \end{array} \end{array}$$ (2)

We assume that the contact rate for all the subpopulations except the infected group is equal to the baseline contact rate, *c*_*B*_, while for the infected subpopulation it is set to *c*_*B*_/2 to reflect the fact that infected people are partially isolated.

The vaccine sharing event determines the respective vaccine stocks in the two countries as functions of time throughout the simulation horizon. We formulate an optimization problem over all possible values of *μ* ∈ [0, 1] with the aim of minimizing the fatalities in country A at the final time, *t*_*f*_. Defining the fatalities in country A at time t as *D*_*A*_(*t*), and the cost function as *J*(*μ*) = *D*_*A*_(*t*_*f*_), the optimization problem is

$$\begin{array}{c} \underset{\mu \in [0,1]}{\text{min}}J\left(\mu \right)≔\underset{\mu \in [0,1]}{\text{min}}D_{A}\left(t_{f}\right). \end{array}$$ (3)

We solve this problem by a dynamic gradient-descent algorithm based on optimal control whose code, written in MATLAB, is available in https://github.com/WeitzGroup/vaccine_allocation.

## Supporting information

S1 Table Parameters and initial conditions used in state Eq (2). In the table above, *i* ∈ {*A*, *B*} and the initial conditions for both countries are the same.
(PDF)

S1 Fig Fatalities in countries A and B when the (a) no-sharing policy, (b) optimal policy and (c) hybrid policy is implemented, over different coupling constants, *κ* ∈ [10^−8^, 10^−1^] and vaccination rates, λ (from 0.14% to 0.42% of the population daily). The optimal policy has the objective of minimizing fatalities in country A and the optimal sharing fraction is μ*. The hybrid policy is a near optimal policy with a sharing fraction of 1/3 when μ* ≤ 1/3 and equal to the optimal sharing fraction otherwise. The optimal policy shows significant reduction in fatalities in B when vaccination rate is low and coupling constant is high, when compared to the no-sharing policy. The hybrid policy results in major reduction in fatalities in B for all vaccination rates and coupling constants, when compared to the no-sharing policy. There is a slight increase in fatalities in A for the hybrid policy when the coupling constant is very small and the vaccination rate is high.
(EPS)

S2 Fig Percentage change in fatalities in countries A and B when comparing the hybrid policy with the optimal policy, over different coupling constants, *κ* ∈ [10^−8^, 10^−1^] and vaccination rates, λ (from 0.14% to 0.42% of the population vaccinated daily). The hybrid policy is a near optimal policy with a sharing fraction of 1/3 when μ* ≤ 1/3 and equal to the optimal sharing fraction otherwise. For country A, an increase in fatalities in the range of [0%, 14%] is observed and contours are made for 0%, 5% and 10% increase in fatalities. For country B, a decrease of fatalities in the range of [0%, 85%] can be seen, with contours plotted for 20%, 40%, 60% and 80% fatality reduction. By relaxing the strict condition of minimizing fatalities in A, the near optimal solution provided by the hybrid policy shows significant fatality reduction in B when compared to the optimal policy. However, this comes at the cost of a small increase in fatalities in A in the regime of low coupling constant and high vaccination rate.
(EPS)
