# Supplementary material for: Vaccine stockpile sharing for selfish objectives
Source: PLOS Glob Public Health. 2022 Dec 5;2(12):e0001312. doi: 10.1371/journal.pgph.0001312 (PMC10021782; doi:10.1371/journal.pgph.0001312)
Supplement: S1 Table — In the table above, i ∈ {A, B} and the initial conditions for both countries are the same. (PDF) [file pgph.0001312.s001.pdf]

Parameter	Meaning	Value
η_I	Measure of disease transmission effectiveness	0.1
T_{inc}	Mean incubation period	4 days
T_{inf}	Mean infectious period	6 days
ϕ	Case fatality ratio of infected cases	0.01
c_B	Baseline potentially infectious contact rate	5/day
$S_i(0)$	Initial susceptible population in country i	$10^7 - 500$
$E_i(0)$	Initial exposed population in country i	0
$I_i(0)$	Initial infected population in country i	500
$R_i(0)$	Initial recovered population in country i	0
$V_i(0)$	Initial vaccinated population in country i	0
$D_i(0)$	Initial fatalities in country i	0
V_0	Total vaccines available to country A	7×10^6
t_f	Time horizon	360 days
